# Faith an Element of Success in Practice

**Published:** 1883-02

**Authors:** 


### Faith an Element of Success in Practice.

Dr. Forthergill, in referring to this subject, utters some very sensible remarks. There is no doubt that many physicians make the most lamentable failures on account of their want of faith in therapeutic measures. The effect of the mind on the body is well known, and when the patient sees a lack of confidence in the physician, he is depressed thereby, and the condition is made worse, instead of better. One of the most successful practitioners in one of our Western cities attributes his success in great measure to the faith he himself has in the effectiveness of drugs properly administered, and the confidence that his faith inspires in his patients. Dr. Fothergill says: "If the medical man speak to the patient with doubtful accents and hesitating utterances, he does not inspire confidence; he really sows distrust. This is the explanation of the successful treatment of a case by one man where another has failed, the remedial measures being much the same. The one carries the patient with him to the restoration of health; the other intensifies a morbid state and tends to make it permanent.

This is a matter too little thought about. Just as a weak, willed medical man fails to do certain patients good; and lack of decision of character unfits a medical man for dealing with emergencies, where the judgment must be prompt and the action energetic; so the therapeutic nihilist, who doubts the efficacy of drugs and leaves them chronic valetudinarians; while in the hands of a enthusiast the cases would soon move onward to a satisfactory termination. There are some men who are doubting

Thomases; there are others who decry what they do not understand and deprecate remedies with whose potency they are unacquainted, who do immeasurable harm to their patients. An eclipse of faith in medicines has now existed some time; but the darkness is beginning to move away, and a return of faith, stronger, firmer, more capable of giving a *raison d'etre* for its existence than in the past is dawning—the daybreak of happier times for those who are stricken down with illness, or crippled in their working power by incapacity in their digestive viscera. This therapeutic nihilism is a passing wave of opinion, a temporary mental state, the end of which is at hand; and the sooner it is over the better for all. The patient's prospects will be brighter, the medical man happier for feeling that the patient has got some 'value received' in return for his outlay. A healthier condition of thought on matters medical will generally obtain; for quacks charlatans, and irregular practitioners of all kinds are to a great extent fostered by the recent want of faith in the medical profession. When a man is sick, what he wishes is to get well; the means to him is a matter of comparative indifference."

